# Fluorination Improves the Electro-Optical Properties of Benzoxazole-Terminated Liquid Crystals in High Birefringence Liquid Crystal Mixtures: Experimental and Theoretical Investigations

**DOI:** 10.3390/molecules28073019

**Published:** 2023-03-28

**Authors:** Ran Chen, Zihao Mao, Zhongwei An, Xinbing Chen, Pei Chen

**Affiliations:** Key Laboratory of Applied Surface and Colloid Chemistry (MOE), International Joint Research Center of Shaanxi Province for Photoelectric Materials Science, Shaanxi Key Laboratory for Advanced Energy Devices, Shaanxi Engineering Laboratory for Advanced Energy Technology, School of Materials Science and Engineering, Shaanxi Normal University, Xi’an 710119, China

**Keywords:** liquid crystal mixture, high birefringence, benzoxazole, large dielectric anisotropy, DFT calculations

## Abstract

Aromatic heterocyclic liquid crystal (LC) materials have received much attention from LC chemists for their high birefringence and large dielectric anisotropy, yet few have reported their properties in LC mixtures. In this work, a series of fluorinated benzoxazole liquid crystal compounds were synthesized to evaluate their electro-optical properties in high birefringence LC mixtures, with the expectation of further establishing the theoretical basis and experimental evidence for their applications in LC photonics. Firstly, the effects of the lateral fluorine substituent positions on the molecular synthetic yield, mesomorphic and solubility properties were comparatively investigated. Afterwards, we focused on the fluorination effects on the core electro-optical properties, including birefringence, dielectric anisotropy and further investigation of the viscoelastic coefficient of high birefringence LC mixtures. Research results showed that the benzoxazole liquid crystal compounds possess low melting points, wide nematic phase intervals and good solubility by appropriate lateral fluorine substitution, which is beneficial to further improve the electro-optical properties of high birefringence LC mixtures. Meanwhile, the theoretical and experimental results corroborate each other to well reveal the structure–property relationship. This study demonstrates that fluorination would promote promising applications of benzoxazole-terminated liquid crystals in emerging LC optical devices.

## 1. Introduction

Liquid crystal (LC) materials are always in the spotlight due to their great prospects for applications in emerging display technologies [1] and ever-changing LC photonics, such as virtual reality display, augmented reality display [2], 3D display [3], phase modulators [4], zoomable lenses [5], fiber optic communication [6], beam steering [7] and tunable terahertz devices [8]. These extensive applications have also presented several performance requirements for the development of LC materials. For example, LC photonics require LCs to possess high birefringence for large phase modulation and fast response time, and large dielectric anisotropy to reduce the operating voltage. Therefore, LC materials with high birefringence (Δ*n*) and large dielectric anisotropy (Δ*ε*) have attracted much attention in the field of LC photonics.

Heterocyclic structures such as benzothiazole [9], benzoxazole [10], oxadiazole [11,12], pyrimidine [13], thiophene [14], pyridine [15], benzofuran [16] and benzimidazole [17] have been employed to construct LC compounds which easily present abundant phase transition behavior and luminescence properties. In recent years, the biphenyl LCs containing fluorinated benzofuran [16] ring have been shown to possess a prominent dielectric anisotropy (~30) and high birefringence (~0.3). The fluorinated benzimidazole LCs [17] have a high melting point of 130.8 °C and a wide smectic phase interval. A novel fluorinated benzothiazole LC [18] showed a wide nematic range and a high melting point (186.9 °C). Among them, the benzoxazole structure is prone to high Δ*n* and large Δ*ε* due to their large π-conjugations and strong dipole–dipole interactions derived from nitrogen and oxygen atoms [19,20]. Despite their high Δ*n* and large Δ*ε*, they suffer from high melting point and wide smectic phase interval to hinder their practical applications. The growing scientific interest in benzoxazole-terminated LC molecules for applications in LC mixtures has inspired us to develop more effective LCs. For example, by optimizing the benzene ring number and the type of mesophase units [21], the LC phase temperature interval was effectively broadened and the melting point was also lowered. The lateral fluorine substituents were successfully utilized to lower the melting point and improve the nematic phase stability by suppressing the smectic phase [22]. The insertion of a carbon–carbon triple bond between two benzene rings increased the π-conjugations and molecular length, which effectively enhanced the Δ*n* and suppressed the smectic phase [23,24]. Therefore, a series of benzoxazole LCs with excellent performance were finally obtained by the above molecular engineering strategies. It is believed that the application of these compounds in high-Δ*n* LC mixtures will be beneficial for fast-response LC photonics.

Based on the above background, a series of fluorinated benzoxazole LCs were synthesized to comparatively study the influence of the lateral fluorine substituent positions on their molecular synthetic yields and thermal properties. Subsequently, we selected the commercial high-Δ*n* LC mixture (HTD028200-100) as the parent formulation to investigate their solubility and their effects on the clearing point of the parent LC mixture. Finally, combined with DFT calculations [25,26,27,28,29,30,31,32], we expect to lay a theoretical foundation for the study of the fluorination effect on the intrinsic properties of these benzoxazole LCs, such as molecular configuration, polarizability, dipole moment and aspect ratio, to correlate the influence of these compounds on the electro-optical properties such as birefringence, dielectric anisotropy and viscoelastic coefficient of high-Δ*n* LC mixture. The electro-optical properties of target fluorinated target compounds (**F_1_**, **F_2_**, **F_3_**, **F_4_**) and their mixtures with commercially available high birefringence LC mixture (**M_1_**, **M_2_**, **M_3_** and **M_4_**) could also be tuned via doping of QDs [33].

## 2. Results and Discussion

### 2.1. Effect of Fluorination on Solubility

**Figure 1:**
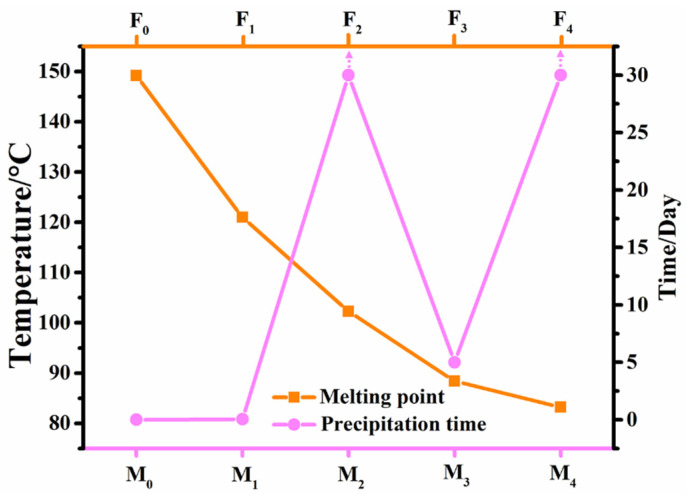
Melting points of benzoxazole LCs and their precipitation times in the parent LC mixture.

In practical applications, many LC compounds need to be used in LC mixtures, hence the solubility is the first thing to be considered. Since some benzoxazole LC compounds exist in the smectic phase, a larger doping amount may destroy the nematic phase stability of the parent LC mixture. Therefore, a mass fraction of 15% was selected for this experiment, and the solubility of compounds **F_0_**–**F_4_** in the parent LC mixture was observed in a follow-up manner, and the recorded results of the precipitation time are shown in Figure 1.

It is obvious from Figure 1 that the non-fluorinated benzoxazole LC compound **F_0_** could not dissolve 15 wt% in the parent LC mixture **M**, while the introduction of the lateral fluorine substituent could effectively enhance the solubility of these benzoxazole LCs in the parent LC mixture. Among them, compounds **F_2_** and **F_4_** had the best solubility performance in the parent mixture and could be stored at room temperature for more than 30 days. The LC mixtures **M_3_** and **M_1_** started to show partial insolubility after 5 days and 1 h of storage time, respectively. According to Equation (1), it is known that the solubility is mainly determined by both the melting point and corresponding melting enthalpy, thereby we firstly correlated their solubility in the parent mixture with their melting points (Table 1). It can be seen that the precipitation times of new LC mixtures, except for the LC mixture **M_2_**, can correspond well to the melting points of their corresponding compounds. For example, compounds **F_0_** and **F_1_** show the highest melting points, thereby the corresponding mixtures **M_0_** and **M_1_** precipitate most readily at room temperature. Comparing compounds **F_2_** and **F_3_**, it can be seen that the lower melting enthalpy of **F_2_** corresponds to a longer precipitation time of the mixture **M_2_**, which may explain the above anomaly. In summary, the introduction of the lateral fluorine substituent lowers the melting point of the LC compound, further enhancing their solubility in the LC mixture, and providing a possibility for studying device performance.

### 2.2. Effect of Fluorination on Mesomorphic Properties

**Figure 2:**
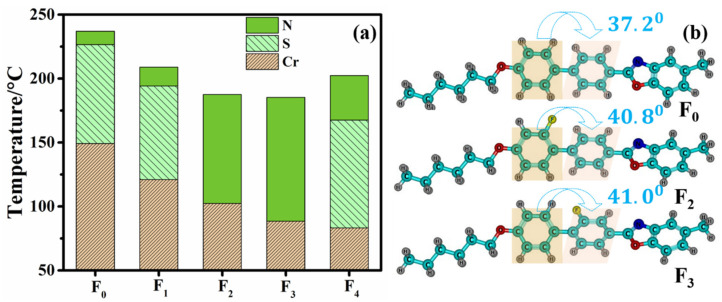
Phase transition temperature (**a**) of benzoxazole LC compounds and biphenyl dihedral angles (**b**) in the molecular configuration of compounds **F_0_**, **F_2_** and **F_3_**.

Different lateral fluorine substitution positions bring about changes in molecular conformation, such as molecular aspect ratio and biphenyl dihedral angle, which possibly modulate the LC phase state and LC phase transition temperature of the LC compounds. Consequently, we tested the thermal properties of compounds **F_0_**–**F_4_** using DSC and POM instruments, and the test results are shown in Figure 2 and Figure S1 (Supplementary Materials). From Figure 2a, it can be seen that the lateral fluorine atom significantly reduces the melting point and clearing point of the benzoxazole LCs, and also changes the LC phase state and LC phase temperature interval. Compared with the previous fluorinated LCs based on benzimidazole [17] and benzothiazole [18], these benzoxazole LCs possessed wider nematic phase intervals and lower melting points. When the lateral fluorine atom is on the inner side of the biphenyl rings (**F_2_** and **F_3_**), the molecular aspect ratio of benzoxazole LCs seems to be unchanged but causes a larger biphenyl dihedral angle. This more distorted spatial steric configuration helps to suppress intermolecular π–π stacking and the appearance of smectic phases, further lowering the melting and clearing points while broadening the nematic phase temperature interval. For example, compared to compound **F_0_**, compounds **F_2_** and **F_3_** have a larger biphenyl dihedral angle (Figure 2b), and thereby exhibit lower melting and clearing points, and a wider nematic phase temperature interval [34,35]. Compared to the non-fluorinated compound **F_0_**, when the lateral fluorine atom is on the outer side of the biphenyl rings (**F_1_** and **F_4_**), their biphenyl dihedral angles are similar, but the molecular aspect ratio is significantly larger (4.4 vs. 4.9), which facilitates the wider LC phase temperature interval. This phenomenon has been reported in a previous paper by Gray [36].

**Figure 3:**
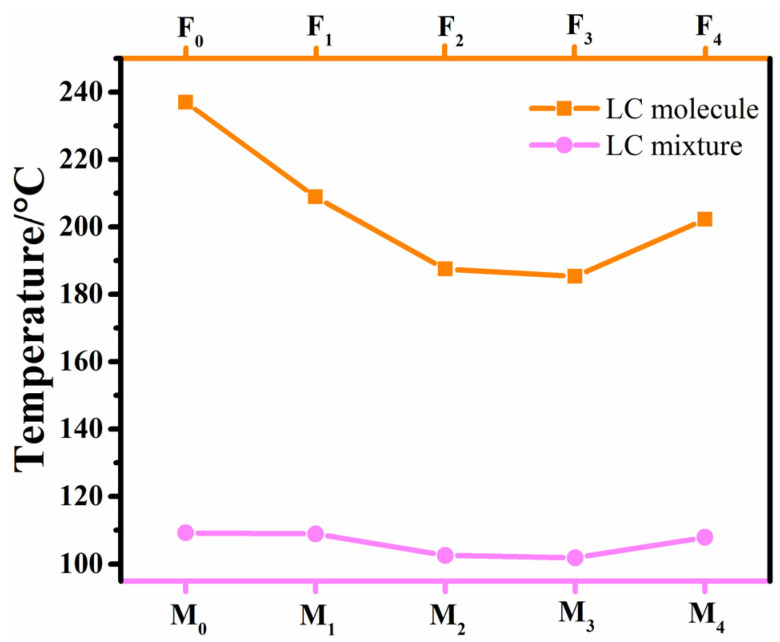
The clearing points of benzoxazole LC compounds and their corresponding LC mixtures.

These LC compounds have high clearing points to be suitable as high-temperature components in LC mixtures. As shown in Figure 3, the clearing point of the parent mixture is 94.5 °C. After adding the benzoxazole LC compounds, the clearing points of LC mixtures **M_0_**–**M_4_** were all significantly increased (>100 °C). After the corresponding LC compounds were blended into the parent mixture **M** with the same mass fraction, the change tendency of the clearing points for the LC mixtures (**M_0_**, **M_1_**, **M_2_**, **M_3_**, **M_4_**) was consistent with that of the corresponding LC compounds. This indicates that the contribution of benzoxazole LC compounds to the clearing point of the parent LC mixture corresponds to the Equation (2). It can also be seen that their additions can effectively enhance the clearing point of the parent LC mixture, which is conducive to the high temperature applications of LC mixtures.

### 2.3. Effect of Fluorination on Birefringence

**Figure 4:**
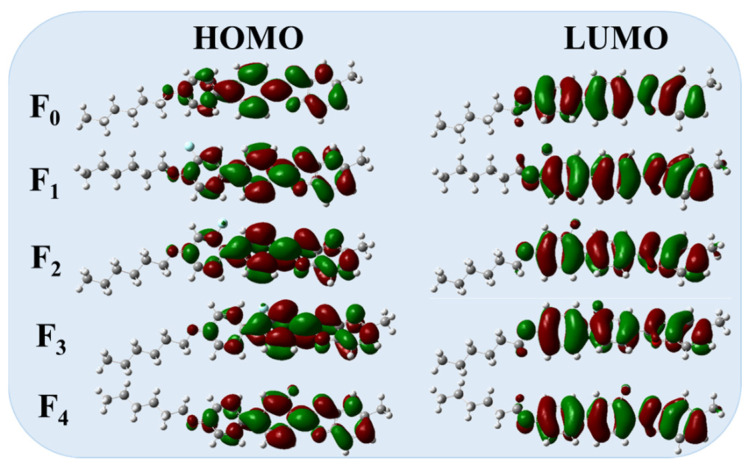
The frontier orbitals and energy levels of these benzoxazole LC compounds by DFT calculations.

**Figure 5:**
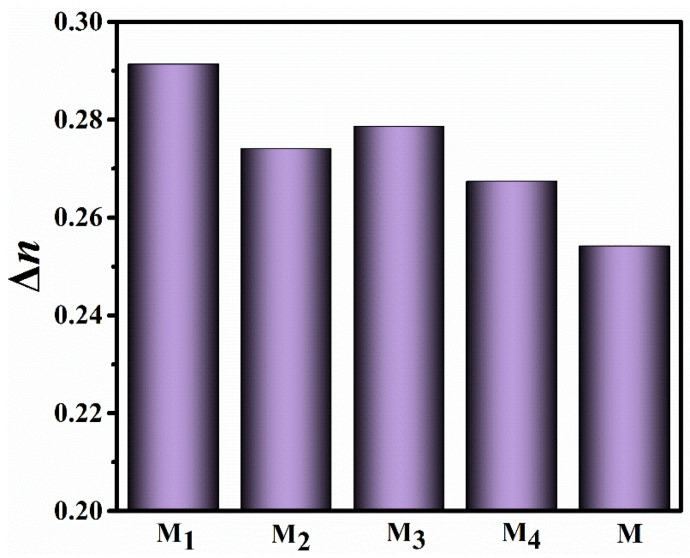
The birefringence values of LC mixtures at 25 °C.

The fluorination brings about changes in frontier molecular orbitals and π-electron density (Figure 4), which maybe affect the π–π conjugations and birefringence of these LC compounds. Herein, we measured the birefringence values of the parent LC mixture **M** and new mixture **M_1_**–**M_4_** using polarized interference method, and the test results are shown in Figure 5. The birefringence of the mixture **M_0_** was not obtained because the non-fluorinated LC compound **F_0_** is extremely easy to precipitate at room temperature.

The test results showed that doping each of these benzoxazole LCs into the parent LC mixture **M** could enhanced its birefringence to a large extent. By extrapolation, the birefringence values of these target compounds are shown in Table 2, and their birefringence values are bigger than those of benzofuran LCs [16]. The birefringence values of the mixture **M_1_**–**M_4_** are arranged in the order: **M_1_** > **M_3_** > **M_2_** > **M_4_**. This indicates that the Δ*n* values are larger when the lateral fluorine atoms are at positions 1 and 3, and the Δ*n* values are smaller when the lateral fluorine atoms are at positions 2 and 4. The influence of benzoxazole LCs on the birefringence of the parent LC mixture is related to its own birefringence, thereby we first investigated the influence of lateral fluorine substituents on the birefringence of benzoxazole LCs from the perspective of theoretical calculations.

According to Vuks’ formula, the birefringence of the LC compounds is closely related to the parameters such as the isotropic component $\overset{-}{\alpha }$, anisotropy Δ*α* and molecular order parameter (*S*) obtained from theoretical calculations (Table 3). Meanwhile, the birefringence values obtained from theoretical calculations are in the following order: **F_4_** > **F_1_** > **F_3_** > **F_2_**. The experimental Δ*n* values of the mixtures **M_1_**–**M_3_** present the same trend of the theoretical Δ*n* magnitudes for compounds **F_1_**–**F_3_**, which indicates that the theoretical calculations can correlate well with the experimental results. Although the theoretical Δ*n* of **F_4_** is the largest, the experimental Δ*n* of the corresponding mixture **M_4_** is the smallest, which may be related to the *S* change of molecule **F_4_** after doping into the parent LC mixture. The interaction between the LC molecule **F_4_** and other LC molecules in the parent LC mixture and the influence of the orientation layer in a LC cell on the LC molecule would lead to the *S* change.

### 2.4. Effect of Fluorination on Dielectric Anisotropy

**Figure 6:**
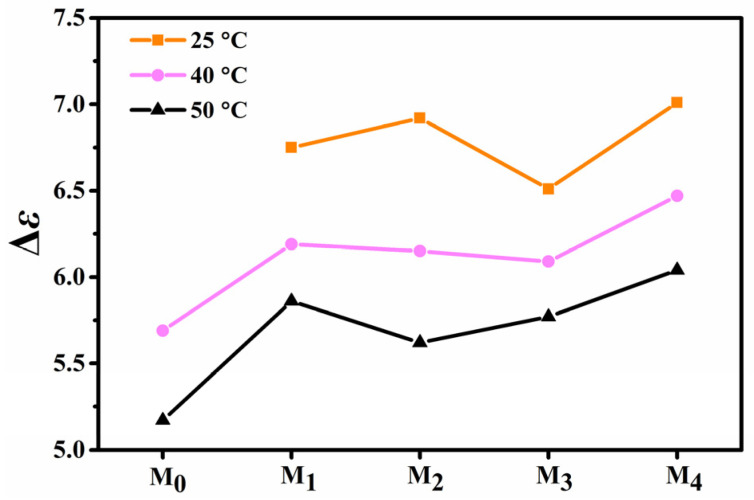
The dielectric anisotropy values of LC mixtures at 25 °C, 40 °C, 50 °C.

In order to compare the dielectric anisotropy of the LC mixtures **M_0_**–**M_4_**, we tested their dielectric anisotropy at 40 °C and 50 °C, and the data are shown in Figure 6. From Figure 6 and Table 3, it can be seen that the lateral fluorine substituent in the benzoxazole LCs increases the dielectric anisotropy of the corresponding LC mixtures **M_1_**–**M_4_**. The observed enhancement in dielectric anisotropy due to fluorination would lead to an increase in rotational viscosity. This will slow down the switching speed of liquid crystal material. The Δ*ε* values of the LC mixtures **M_2_** and **M_4_** are larger when the lateral fluorine substituents are at position 2 and position 4. The Δ*ε* values of the LC mixtures **M_0_**–**M_4_** decrease as the test temperature increases, which is due to the decrease in the molecular order parameter caused by the increase in temperature. The relative magnitudes of Δ*ε* values of LC mixtures, except **M_2_**, follow the same trend when the test temperature increases. This indicates that the Δ*ε* of mixture **M_2_** is more sensitive to temperature changes, which may be related to the lower clearing point of the LC mixture **M_2_**.

The effect of the benzoxazole LCs on the Δ*ε* of the parent LC mixture is related to its own Δ*ε*. And the contribution of one LC compound to the Δ*ε* of the parent LC mixture corresponds well to the Equation (3), when the addition of the LC compound does not affect the molecular arrangement of other LC molecules in the LC cell to a large extent. Therefore, we first investigated the effect of the lateral fluorine substituent on the Δ*ε* of the benzoxazole LCs from the perspective of theoretical calculations. According to the Maier and Meier formula, it is known that the Δ*ε* of the LC compound is closely related to the molecular dipole moment (*μ*), the anisotropy polarizability (∆*α*), the angle (θ) between the permanent dipole moment and molecular long axes. From the viewpoint of theoretical calculations, the molecular dielectric anisotropy depends mainly on its molecular dipole moment, hence we use the effective dipole moment to correlate the experimental and theoretical results. The effective dipole moments of compounds **F_1_**–**F_4_** are arranged in the order: **F_4_** > **F_2_** > **F_1_** > **F_3_**, and the Δ*ε* values for the corresponding LC mixtures **M_1_**–**M_4_** at room temperature show the same change trend, which indicates that the theoretical calculations can be well correlated with the experimental results (Table 4).

### 2.5. Effect of Fluorination on Viscoelastic Constant

**Figure 7:**
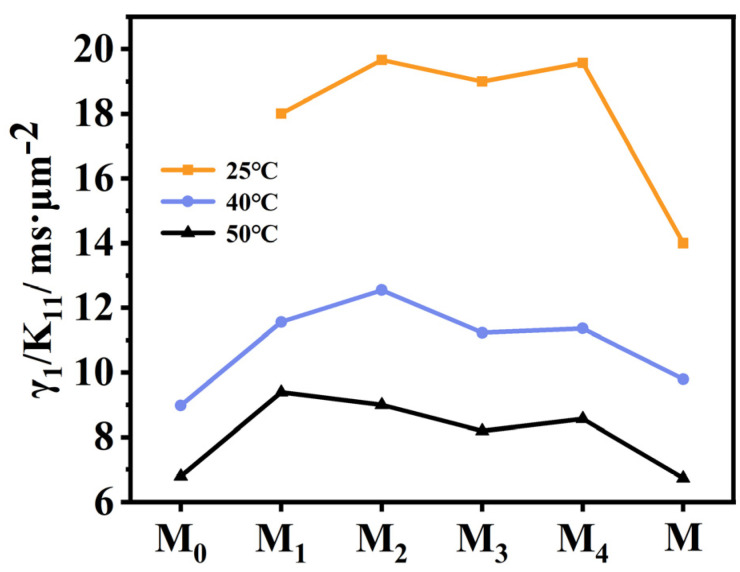
The viscoelastic constants of LC mixtures at 25 °C, 40 °C and 50 °C.

Fast response speed of liquid crystals is mainly associated with their low viscoelastic coefficient and high Δ*n* properties. Therefore, we tested the viscoelastic constants (*γ*_1_/*K*_11_) of six LC mixtures **M**–**M_4_** at 25 °C, 40 °C and 50 °C, and the data were shown in Figure 7. From Figure 7, it can be seen that the *γ*_1_/*K*_11_ values of new LC mixtures all increase significantly when each of these benzoxazole LCs is added to the parent LC mixture at room temperature. Among the four LC mixtures **M_1_**–**M_4_**, it can be seen that the *γ*_1_/*K*_11_ values of the LC mixtures **M_2_** and **M_4_** are larger than those of **M_1_** and **M_3_** at room temperature, which may be related to the fact that LC compounds **F_2_** and **F_4_** possess strong dipole–dipole interactions. Generally, the stronger the dipole–dipole interaction is, and thus the larger the rotational viscosity is, the larger the *γ*_1_/*K*_11_ value is. Furthermore, when the lateral fluorine substitution is on the inner side of the biphenyl rings (**F_2_** and **F_3_**), the corresponding LC mixture **M_2_** or **M_3_** tends to show a larger viscoelastic constant, which may be related to its lower clearing point according to Equation (4).

When the device temperature is 40 °C, the viscoelastic constants of the LC mixtures **M_1_**–**M_4_** containing the fluorinated LC compounds **F_1_**–**F_4_** and the parent LC mixture **M** are larger when compared to the LC mixture **M_0_** containing the non-fluorinated LC compound **F_0_**. Among the LC mixtures **M_1_**–**M_4_**, since the LC mixture **M_1_** has the highest clearing point, its viscoelastic constant decreases more slowly with increasing temperature, finally presenting a larger viscoelastic constant at 40 °C and 50 °C. Similarly, the LC mixture **M_3_** has the smallest clearing point, whereby its viscoelastic constant decreases rapidly with increasing temperature. This indicates that the increase in device temperature is more beneficial for reducing the viscoelastic constants of LC mixtures **M_2_** and **M_3_**, and further improving the device response time.

## 3. Materials and Methods

### 3.1. Materials

The key raw materials, such as 4-bromophenol, 2-fluoro-4-bromophenol, 3-fluoro-4-bromophenol, 4-formylphenylboronic acid, 2-fluoro-4-formylphenylboronic acid and 3-fluoro-4-formylphenylboronic acid, were purchased from Aladdin-reagent Co. (Shanghai, China) and used as received. Other reagents and solvents were purchased from Sinopharm Chemical Reagent Co. (Shanghai, China) High birefringence LC mixture HTD028200-100 was purchased from the Jiangsu Hecheng Display Technology Co., Ltd. (Nanjing, China). Liquid crystal cells with a thickness of 5 µm were purchased from the Northern Liquid Crystal Engineering Research and Development Centre (Changchun, China).

### 3.2. Synthesis of Target Compounds

**Figure 8:**
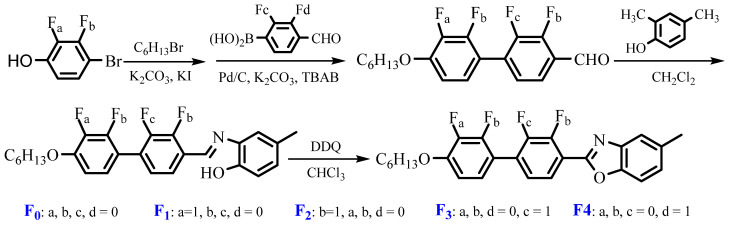
Synthesis route of fluorinated benzoxazole LC compounds.

The target compounds were synthesized in three steps using the classical reactions of Williamson, Suzuki Coupling, Schiff base and subsequent intramolecular cyclisation reactions, and the synthetic routes of the five benzoxazole LC compounds are shown in Figure 8. **F**_0_ (2-(4′-hexyloxybiphenyl)-5-methylbenzoxazole) was chosen as the reference compound. **F_1_** (2-(3′-fluoro-4′-hexyloxybiphenyl)-5-methylbenzoxazole), **F_2_** (2-(2′-fluoro-4′-hexyloxybiphenyl)-5-methylbenzoxazole), **F_3_** (2-(2-fluoro-4′-hexyloxybiphenyl)-5-methylbenzoxazole), **F_4_** (2-(3-fluoro-4′-hexyloxybiphenyl)-5-methylbenzoxazole) correspond to show lateral fluorine substituent at positions 1, 2, 3 and 4 in biphenyl rings, respectively. The lateral fluorine substituent positions have a great influence on their synthesis conditions as well as their synthetic yields. For example, when the lateral fluorine substituent was at position 2, the synthetic yield was about 20%, while the yield was elevated to 46% when the lateral fluorine substituent was at position 3. The HPLC purities of target compounds were all above 98%, and their chemical structures were confirmed by ^1^H NMR, IR and mass spectra. The synthesis procedures and characterized data of target compounds were provided in the Supplementary Materials.

### 3.3. The Preparation of LC Mixtures and Solubility Experiments

In order to fully investigate the optoelectronic properties of LCs in LC mixtures, it is required that the mass fraction of LC compounds blended into the LC mixture is as large as possible. The target compounds **F_0_**–**F_4_** were added into commercial high-∆*n* LC mixture (HTD028200-100, noted as M) with the mass fraction of 15%, respectively, to prepare five new LC mixtures (**M_0_**, **M_1_**, **M_2_**, **M_3_**, **M_4_**). The solubility experiments were determined by standing the newly prepared LC mixtures at room temperature, observing whether the precipitation appears, and then recording the precipitation time. Initially, the LC mixture can maintain the original state without precipitation, after a certain period of time (t_1_), the precipitation phenomenon appears. The precipitation time refers to t_1_. According to the literature [17], the solubility of LC compound in the LC mixture is described by Equation (1), where A is the activity coefficient, *ΔH*_1_ is the enthalpy change of LC compound corresponding to the melting point *T*_1_, *T* is the measured temperature, and *X*_1_ is the molar fraction of the LC compound.

(1) $$\ln(A\cdot X_{1})=-\frac{\Delta H_{1}}{R}(\frac{1}{T}-\frac{1}{T_{1}})$$

### 3.4. Characterization and Measurements

The molecular structures of the target compounds and intermediates were determined by Nuclear magnetic resonance spectroscopy (^1^H NMR) (ECZ400R/S1, Nippon Electronics Co., (Tokyo, Japan), Fourier transform infrared spectrometer (IR) (FTIR Tensor27, Brucker, Germany), gas phase mass spectrometry (GC-MS) (DSQ II, Thermo Fisher Scientific Co., Ltd., Waltham, MA, USA). The mesomorphic properties of target compounds **F_0_**–**F_4_** were tested by differential scanning calorimeter (DSC) and polarizing optical microscope (POM). DSC test conditions: under the protection of nitrogen, the heating and cooling rates are both 5 °C /min. The LC texture can be confirmed by POM and previous reported texture. The above characterized process is shown in the supporting information. The Δ*n* values of LC mixtures (**M_0_**, **M_1_**, **M_2_**, **M_3_**, **M_4_**) were calculated from the measured phase retardation at 25 °C [31]. The viscoelastic constant (*γ*_1_/*K*_11_) values of all LC mixtures were calculated through transient current method by autronic-MELCHRS LCCS107 [37]. The dielectric anisotropy (∆*ε*) of all LC mixtures were measured with a multifrequency LCR meter IM-3536. If not otherwise specified, all of the measurements for LC mixtures were carried out at temperatures of 25 °C, 40 °C, 50 °C, frequency of 1 kHz and wavelength of 633 nm.

Equations (2) and (3) can be used to deduce the clearing points (*T*_c_) and ∆*ε* values of the new LC mixtures, which are extrapolated by the host–guest method [38].

(2) $$T_{c}=xT_{c}^{2}+(1-x)T_{c}^{H}$$

(3) $$∆\varepsilon =x{∆\varepsilon }^{2}+(1-x){∆\varepsilon }^{H}$$

where *x* refers to the doping concentration of LC compound, *T*_c_^2^ and ∆*ε*^2^ are the extrapolation values of clearing point and dielectric anisotropy, and *T_c_^H^* and ∆*ε^H^* are the extrapolation values of clearing point and dielectric anisotropy of the parent LC mixture. The temperature-dependent viscoelastic coefficient of LC mixtures can be described by the following equation [39]:

(4) $$\frac{\gamma_{1}}{K_{11}}=A\frac{exp\left(E_{a}/k_{B}T\right)}{{\left(1-T/T_{c}\right)}^{\beta }}$$

In Equation (4), where A, *k_B_* and *E_a_* represent the proportionality constant, Boltzmann constant, and activation energy, respectively.

### 3.5. DFT Calculations

Based on previous theoretical research [25,26,27,28,29,30,31,32] on liquid crystals, it is known that molecular inherent parameters such as the polarizability, dipole moment, aspect ratio and biphenyl dihedral angle are closely related to the properties of LCs such as birefringence, dielectric anisotropy and liquid crystal phase transition. Therefore, we used density functional theory (DFT) to optimize the geometric configuration of the LC compounds **F_0_**–**F_4_**, and the relevant theoretical calculations obtained were used to correlate the relevant experimental results. Gaussian 09 software was employed to carry out DFT calculations of our target compounds. Further, the frequency calculations were performed to ascertain whether the optimized geometry was achieved. The Vuks formula [40] is as follows:

(5) $$\frac{{n_{e}}^{2}-1}{n^{2}+2}=\frac{N}{3\varepsilon_{0}}\left[\overset{-}{\alpha }\right.+\frac{2\Delta \alpha \cdot S}{3}\left.\right]$$

(6) $$\frac{{n_{o}}^{2}-1}{n^{2}+2}=\frac{N}{3\varepsilon_{0}}[\overset{-}{\alpha }-\frac{\Delta \alpha \cdot S}{3}]$$

Maier and Meier formula [41] is as follows:

(7) $$∆\varepsilon =\mathrm{NhFS}\left\{∆\alpha \right.-F\frac{\mu^{2}}{2KT}\left(1-3\cos^{2}\left.\theta \right)\right.\left.\right\}$$

## 4. Conclusions

Quantum chemical calculation is an efficient tool to assist in guiding the design and synthesis of LC compounds with excellent performance. In this work, a series of fluorinated benzoxazole-terminated LCs were synthesized to comparatively investigate the fluorination effects on solubility and mesomorphic properties, and to further explore their effects on the electro-optical properties of high birefringence LC mixtures. Combined with DFT calculations, the research results of the structure–property relationship are as follows.

The lateral fluorine substituents can increase the molecular aspect ratio and biphenyl dihedral angle, further weakening the intermolecular π–π stacking and intermolecular interactions, resulting in lower melting and clearing points, disappearance of the smectic phase, wide LC phase temperature range and good solubility. The lateral fluorine substituents change the isotropic component $\overset{-}{\alpha }$, anisotropy polarizability and order parameter of the molecules to obtain the numerical order of the theoretical birefringence by: **F_4_** > **F_1_** > **F_3_** > **F_2_**, which can well explain the numerical relationship of the birefringence for the corresponding LC mixtures **M_1_**–**M_3_**. Correlating the effective dipole moment with dielectric anisotropy of LC compounds, it is found that the theoretical effective dipole moments of compounds **F_1_**–**F_4_** have the same variation trend as the experimental dielectric anisotropy of the corresponding LC mixtures **M_1_**–**M_4_**. Through a suitable fluorination strategy, the corresponding LC mixtures can obtain high clearing points, high birefringence, large dielectric anisotropy, and viscoelastic constants with temperature-sensitive variation. This means that fluorination would promote promising applications of benzoxazole-terminated liquid crystals in emerging LC optical devices.

## Figures and Tables

**Table 1:** Types of mesophases, transition temperatures and corresponding enthalpies obtained by POM and DSC methods for compounds **F_0_**–**F_4_**.

Compound	Transition Temperature/°C (Enthalpy Change/kJ mol^−1^)	
	Heating Process ^a^	Cooling Process ^a^
**F_0_**	Cr 149.2 (7.6) SmC 226.6 (3.90) N 237.0 (0.90) I	I 234.7 (−1.00) N 224.2 (−3.70) SmC 144.6 (−7.20) Cr
**F_1_**	Cr 121.0 (23.25) SmC 194.2 (1.75) N 208.9 (0.76) I	I 207.3 (−0.89) N 192.5 (−1.47) SmC 85.4 (−19.90) Cr
**F_2_**	Cr 102.3 (24.40) N 187.5 (0.60) I	I 186.4 (−0.54) N 79.5 (−20.50) Cr
**F_3_**	Cr 88.4 (25.94) N 185.3 (0.55) I	I 185.5 (−0.67) N 75.8 (−22.93) Cr
**F_4_**	Cr 83.2 (24.11) SmC 167.5 (1.38) N 202.3 (0.73) I	I 201.0 (−0.84) N 166.2 (−1.00) SmC 48.1 (−9.22) Cr

^a^ Cr: crystal; SmC: smectic C mesophase; N: nematic mesophase; I: isotropic liquid.

**Table 2:** Dielectric anisotropy and the corresponding parallel and perpendicular electric primitivities of LC mixtures **M_1_**–**M_4_** at 25 °C.

LC Mixture	M_1_	M_2_	M_3_	M_4_
*ε*_‖_	9.77	10.08	9.62	9.93
*ε*_⊥_	3.02	3.16	3.11	2.92
Δ*ε*	6.75	6.92	6.51	7.01
Δ*ε*′	4.28 ^a^	5.43 ^a^	2.68 ^a^	6.03 ^a^
Δ*n*′	0.50 ^a^	0.39 ^a^	0.42 ^a^	0.34 ^a^

^a^ Δ*ε*′ and Δ*n*′ refer to the extrapolated dielectric anisotropy and birefringence of the corresponding LC compounds **F_1_**–**F_4_**, respectively.

**Table 3:** The DFT calculated isotropic component $\overset{-}{\alpha }$ = (*α*_XX_ + *α*_YY_ + *α*_ZZ_)/3, anisotropy Δ*α* = [*α*_XX_ − (*α*_YY_ + *α*_ZZ_)/2], and theoretical birefringence (Δ*n*_1_) of target compounds ^a^.

Compound	F_0_	F_1_	F_2_	F_3_	F_4_
*α*_XX_	649.96	645.16	641.99	648.08	653.64
*α*_YY_	265.65	262.84	264.39	264.65	266.15
*α*_ZZ_	164.51	168.71	166.47	166.33	164.33
$\overset{-}{\alpha }$	360.04	358.90	357.62	359.69	361.38
Δ*α*	434.88	429.39	426.56	432.59	438.40
Δ*n*_1_	0.4958	0.4566	0.4446	0.4499	0.4634

^a^ All polarizability components and the anisotropy parameter are expressed in Bohr^3^ (with 1 Bohr = 0.52917 Å).

**Table 4:** The DFT calculated anisotropy Δ*α* = [*α*_XX_ − (*α*_YY_ *+ α*_ZZ_)/2], dipole moment (*µ*) and molecular dipole moment at long axis (*µ*_x_) of target compounds.

Compound	F_0_	F_1_	F_2_	F_3_	F_4_
*µ* (Debye)	2.8938	1.8148	4.0915	3.3497	3.1741
*µ*_x_ (Debye)	−2.2189	−0.7043	2.8062	1.4570	−2.8422
Cos^2^θ ^a^	0.59	0.15	0.47	0.19	0.80
Δ*α*	434.88	429.39	426.56	432.59	438.40
*µ*′ (Debye) ^b^	6.45	−1.81	6.86	−4.82	14.10

^a^ θ is the angle between the permanent dipole moment and the direction of the molecular long axis. ^b^ Effective dipole moment *µ*′ = −*µ*^2^(1 − 3cos^2^θ).

## Data Availability

The data presented in this study are available in this article, and from authors upon request.

## Supplementary Materials

The following supporting information can be downloaded at: https://www.mdpi.com/article/10.3390/molecules28073019/s1, Synthesis and characterized data of target compounds, Figure S1: DSC traces and POM images of **F_2_** and **F_3_**.
