# Supplementary material for: Fluorination Improves the Electro-Optical Properties of Benzoxazole-Terminated Liquid Crystals in High Birefringence Liquid Crystal Mixtures: Experimental and Theoretical Investigations
Source: Molecules. 2023 Mar 28;28(7):3019. doi: 10.3390/molecules28073019 (PMC10096087; doi:10.3390/molecules28073019)
Supplement: Supplementary file 1 [file molecules-28-03019-s001.zip › molecules-2274217-supplementary.pdf]

Supplementary Materials

Fluorination Improves the Electro-Optical Properties of Benzoxazole-Terminated Liquid Crystals in High Birefringence Liquid Crystal Mixtures: Experimental and Theoretical Investigations

Ran Chen,* Zihao Mao, Zhongwei An, Xinbing Chen,* and Pei Chen

Key Laboratory of Applied Surface and Colloid Chemistry (MOE), International Joint Research Center of Shaanxi Province for Photoelectric Materials Science, Shaanxi Key Laboratory for Advanced Energy Devices, Shaanxi Engineering Laboratory for Advanced Energy Technology, School of Materials Science and Engineering, Shaanxi Normal University, Xi'an 710119, China

* Correspondence: tradchenr@snnu.edu.cn (R. C.); chenxinbing@snnu.edu.cn (X. C.).

1. Synthesis and characterized data of target compounds

The synthesis process of compound **F_2** is described below as an example.

1.1. Synthesis of 4-hexyloxy-2-fluoro-(1,1′-biphenyl)- 4′-carboxaldehyde

A mixture of 3-fluoro-4-bromophenol (6 g, 31.4 mmol), *n*-hexyl bromide (5.71 g, 34.6 mmol), anhydrous potassium carbonate (13 g, 94.2 mmol), KI (0.52 g, 3.14 mmol) and DMF (80 mL) was stirred for 3 h at 100 °C. After cooling the mixture to room temperature, then adding 4-formylphenyl boronic acid (5.18 g, 34.6 mmol), Pd/C (0.35 g, 0.3 mmol), tetrabutylammonium bromide (0.51 g, 1.6 mmol), and water (20 mL) to the above system under N_2 protection. Then, the reaction system was further stirred at 100 °C for another 5 h. After the reaction was completed, it was extracted with dichloromethane and dried over anhydrous magnesium sulfate to obtain organic residue.

Finally, white crystals (3.10 g) were obtained by drying at 45 °C in a vacuum oven. The above organic residue was purified using PE/EA (50/1) as eluent to obtain HPLC purity of 98% and yield of 30%.

1.2. Synthesis of 2-(((2′-fluoro-4′-hexyloxy-1,1′-biphenyl-4-yl)methylene) amino)-4-methylphenol

In a 100 mL three-neck round bottom flask, a mixture of 4-hexyloxy-2-fluoro-(1,1′-biphenyl)-4′-carboxaldehyde (0.66 g, 2.20 mmol), 2-amino-4-methyl phenol (0.32 g, 2.67 mmol) and dichloromethane (25 mL) was stirred under reflux condition for 6 h. The solvent was removed under vacuum and recrystallized with methanol to obtain a yellow crystal (0.75 g) with HPLC purity above 98% and yield of 84%. The characteristic data are as follows.^{1}H NMR (300 MHz, $CDCl_3$): δ (ppm) 8.72 (s, 1H), 7.96 (d, *J* = 8.4 Hz, 2H), 7.64 (d, *J* = 6.9 Hz, 2H), 7.40 (t, *J* = 8.7 Hz, 1H), 7.12 (d, *J* = 9.6 Hz, 2H), 7.02 (m, 1H), 6.92 (m, 1H), 6.76 (m, 2H), 4.00 (t, *J* = 6.6 Hz, 2H), 2.33 (s, 3H), 1.80 (m, 2H), 1.39 (m, 6H), 0.92 (t, *J* = 6.9 Hz, 3H). IR (KBr, pellet, cm^{-1}): 3412, 3031, 2953, 2919, 2863, 1615, 1499, 1463, 1401, 1321, 1279, 1227, 1175, 1114, 1020, 960, 842, 808, 740, 626, 580, 526. EI-MS m/z (rel. int.): 405.34 (M^+, 100), 320.21 (37), 188.12 (19), 134.09 (60).

1.3. Synthesis of (2-(2'-fluoro-4'-hexyloxybiphenyl)-5-methylbenzoxazole) (F_2)

A mixture of DDQ (0.33 g, 1.43 mmol), 2-(((2′-fluoro-4′-hexyloxy-1,1′-biphenyl-4-yl) methylene) amino)-4-methylphenol (0.49 g, 1.19 mmol), and anhydrous chloroform (25 mL) was stirred under reflux condition for 6 h. After the reaction mixture was cooled to room temperature, it was extracted with chloroform and dried with anhydrous magnesium sulfate. The solvent was removed under vacuum and recrystallized with ethanol to obtain white crystals (0.42 g) with HPLC purity above 98% and yield of 86%. Its characterized data are as follows. ^{1}H NMR (300 MHz, $CDCl_3$): δ (ppm) 8.28 (d, *J* = 8.7 Hz, 2H), 7.68 (m, 2H), 7.56 (s, 1H), 7.43 (m, 2H), 7.17 (d, *J* = 6.9 Hz, 1H), 6.76 (m, 2H), 4.00

(t, *J* = 6.6 Hz, 2H), 2.50 (s, 3H), 1.81 (m, 2H), 1.42 (m, 6H), 0.92 (t, *J* = 6.9 Hz, 3H). IR (KBr, pellet, cm^{-1}): 3093, 2943, 2865, 1757, 1685, 1611, 1573, 1485, 1403, 1317, 1259, 1229, 1167, 1118, 1060, 1030, 958, 838, 798, 750, 706, 632, 590, 526. EI-MS m/z (rel. int.): 403.31 (M^+, 40), 319.21 (100), 290.20 (8), 213.13 (7), 78.07 (6).

A similar procedure was utilized to prepare the other target compounds $\mathbf{F_0}$, $\mathbf{F_1}$, $\mathbf{F_3}$ and $\mathbf{F_4}$. The other corresponding spectra data are listed as follows:

$\mathbf{F_0}$**:** white solid, yield of 75 %. ^{1}H NMR (300 MHz, $CDCl_3$): δ(ppm) 8.28 (d, *J* = 8.1 Hz, 2H), 7.71 (d, *J* = 8.1 Hz, 2H), 7.60 (d, *J* = 8.4 Hz, 2H), 7.56 (s, 1H), 7.46 (d, *J* = 8.4 Hz, 1H), 7,16 (d, *J* = 8.7 Hz, 1H), 7.00 (d, *J* = 8.4 Hz, 2H), 4.08 (t, *J* = 6.6 Hz, 2H), 2.49 (s, 3H), 1.83 (m, 2H), 1.45 (m, 2H), 1.36 (m, 4H), 0.94 (t, *J* = 6.9 Hz, 3H). IR (KBr, pellet, cm^{-1}): 3087, 2931, 2858, 1612, 1591, 1577, 1541, 1463, 1431, 1382, 1292, 1247, 1174, 1116, 1024, 962, 835, 802, 727. EI–MS m/z (rel. int.): 385.37 (M^+, 52), 301.26 (100), 272.31 (12), 195.13 (7), 77.12 (5).

$\mathbf{F_1}$: white solid, yield of 75 %. ^{1}H NMR (300 MHz, $CDCl_3$): δ (ppm) 8.22 (d, *J* = 8.4 Hz, 2H), 7.62 (d, *J* = 8.4 Hz, 2H), 7.49 (s, 1H), 7.40-7.28 (m, 3H), 7.11 (q, *J* = 8.4 Hz, 0.9 Hz, 1H), 6.99 (t, *J* = 8.4 Hz, 1H), 4.03 (t, *J* = 6.6 Hz, 2H), 2.42 (s, 3H), 1.82 (m, 2H), 1.45 (m, 2H), 1.31 (m, 4H), 0.87 (t, *J* = 6.9 Hz, 3H). IR (KBr, pellet, cm^{-1}): 3051, 2948, 2927, 2859, 1604, 1569, 1550, 1521, 1496, 1470, 1430, 1403, 1307, 1292, 1279, 1265, 1242, 1185, 1133, 1062, 1032, 1013, 939, 847, 809, 744, 701, 524. EI-MS m/z (rel. int.): 403.29 (M^+, 16), 319.19 (100), 290.14 (5), 213.11 (5), 78.03 (4).

$\mathbf{F_3}$**:** white solid, yield of 87 %. ^{1}H NMR (300 MHz, $CDCl_3$): δ (ppm) 8.03 (m, 2H), 7.57 (t, *J* = 8.4 Hz, 4H), 7.47 (d, *J* = 8.1 Hz, 1H), 7.18 (d, *J* = 8.1 Hz, 1H), 7.00 (d, *J* = 8.4 Hz, 2H), 4.02 (t, *J* = 6.6 Hz, 2H), 2.50 (s, 3H), 1.82 (m, 2H), 1.39 (m, 6H), 0.92 (t, *J* = 6.6 Hz, 3H). IR (KBr, pellet, cm^{-1}): 3036, 2943, 2861, 1603, 1550, 1478, 1433, 1402, 1334, 1300, 1249, 1177, 1118, 1059, 1029, 996, 942, 883, 817, 721, 647, 579, 539. EI-MS m/z (rel. int.): 403.35 (M^+, 30), 319.23 (100), 290.22 (10), 213.15 (6), 78.09 (3).

$\mathbf{F_4}$**:** white solid, yield of 58 %. ^{1}H NMR (300 MHz, $CDCl_3$): δ (ppm) 8.29 (t, *J* = 8.1 Hz, 1H), 7.62 (s, 1H), 7.60 (d, *J* = 8.7 Hz, 2H), 7.52 (m, 3H), 7.21 (d, *J* = 8.1 Hz, 1H), 7.01 (d, *J* = 8.7 Hz, 2H), 4.04 (t, *J* = 6.6 Hz, 2H), 2.50 (s, 3H), 1.86 (m, 2H), 1.49 (m, 2H), 1.37 (m, 4H), 0.94 (t, *J* = 6.3 Hz, 3H). IR (KBr, pellet, cm^{-1}): 3045, 2927, 2858, 1742, 1688, 1607, 1574, 1526,

1468, 1435, 1397, 1301, 1245, 1178, 1126, 1027, 881, 812, 694, 527. EI-MS m/z (rel. int.): 403.35 (M^+, 33), 319.25 (100), 290.23 (7), 213.18 (7), 78.06 (4).

2. Characterization processes of spectra, DSC and POM

The 10 mg sample was dissolved in a 0.5 mL deuterium generation reagent ($CDCl_3$), then tested using the ^1H NMR instrument (ECZ400R/S1, Nippon Electronics Corporation, Japan). After drying the KBr in a vacuum oven with 120 °C for 24 h, we mixed a small amount of dried sample and KBr to press into a thin slice, then tested it using the IR instrument (FTIR Tensor27, Brucker, Germany). We dissolved an appropriate amount of the sample in the dichloromethane solution to be tested with the GC-MS instrument (DSQ II, Thermo Fisher Scientific Co., Ltd.). Under the nitrogen protection, a 1–2 mg sample was prepared into a tested cell, then tested with the DSC instrument (DSC-60, Shimadzu Corporation, Japan) at the heating/cooling rate of 5 °C/min. The tested sample was placed in a slide and placed on a Linkam THMSE600 hot stage (Linkam Scientific Instruments Ltd., Tadworth, UK), we then observed the liquid crystal texture with the polarizing microscope (LEICA DM2500P, Leica Microsystems GmbH, Germany) during the heating/cooling rate of 2 °C /min.

3. DSC curves and textures of representative compounds F_2 and F_3

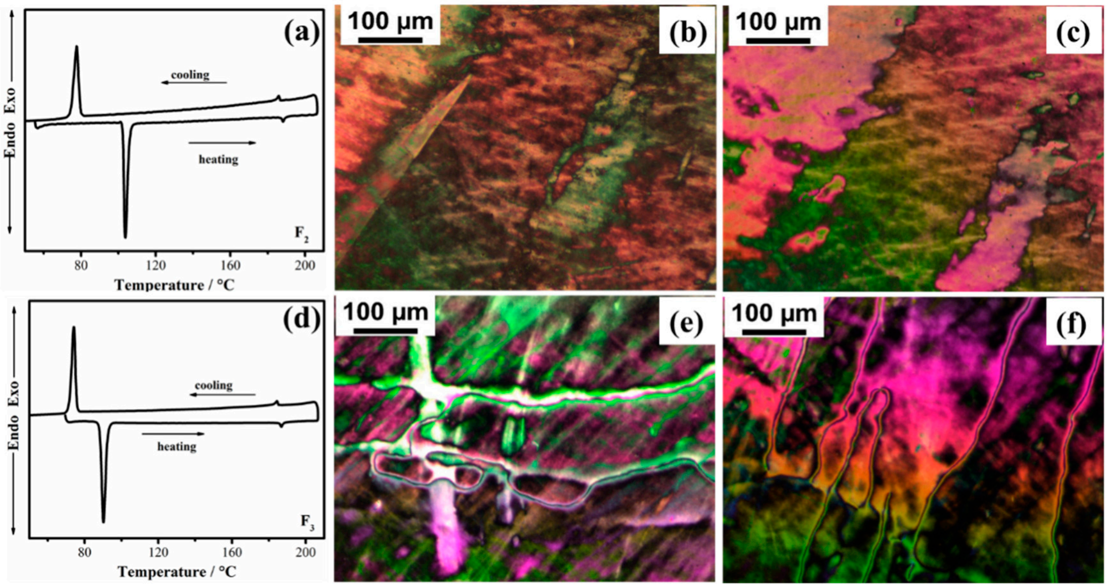

Figure S1. DSC traces of $\mathbf{F_2}$ (a) and $\mathbf{F_3}$ (d), POM images: marble textures of the nematic mesophase for $\mathbf{F_2}$ at 110 °C in the heating scan (b) and 180 °C in the cooling scan (c); marble textures of the nematic mesophase for $\mathbf{F_3}$ at 180 °C in the heating scan (e) and 184 °C in the cooling scan (f).
